# Real world effectiveness of digital mental health services during the COVID-19 pandemic

**DOI:** 10.1371/journal.pone.0272162

**Published:** 2022-08-18

**Authors:** Maximo R. Prescott, Sara J. Sagui-Henson, Camille E. Welcome Chamberlain, Cynthia Castro Sweet, Myra Altman

**Affiliations:** 1 Modern Health, San Francisco, California, United States of America; 2 Clinical Excellence Research Center, Stanford School of Medicine, Palo Alto, California, United States of America; Yamaguchi University: Yamaguchi Daigaku, JAPAN

## Abstract

**Background:**

The COVID-19 pandemic created an unprecedented need for mental health services that can be remotely delivered. Digital mental health services that offer personalized care recommendations hold promise to efficiently expand service, but evidence of the effectiveness of digitally delivered mental health care in real-world settings remains limited.

**Methods:**

A retrospective cohort of adults (*N* = 1,852) receiving care through a digital mental health platform with elevated depressive symptoms during the COVID-19 pandemic was analyzed to estimate changes in subjective well-being and clinical improvement in depressive symptoms (using the World Health Organization-Five [WHO-5] Well-Being Index), as well as compare the relative effectiveness and cost of different care utilization patterns.

**Results:**

The average improvement in WHO-5 score was 10.1 points (CI: 9.3–10.9, p<0.001) at follow-up, which constituted a medium effect size (*d* = 0.73). The odds of clinical improvement in depressive symptoms were significantly greater among those who utilized telecoaching (aOR = 2.45, 95%CI: 1.91–3.15, p < .001), teletherapy (aOR = 2.01, 95%CI: 1.57–2.57, p < .001), and both services (aOR = 2.28, 95%CI: 1.67–3.11, p < .001) compared to those who only utilized assessments, adjusting for baseline WHO-5 score, age, sex, and number of days between baseline and follow-up assessments. The average estimated cost of care for telecoaching was $124 per individual, which was significantly less than teletherapy ($413) or both services ($559).

**Conclusion:**

Digitally delivered care with a therapist and/or coach was effective in improving subjective well-being and clinical improvement in depressive symptoms. Although clinical outcomes were similar across utilization patterns, the cost of care was lowest among those utilizing telecoaching.

## Introduction

The prevalence of mental health conditions continues to outpace the provision of timely, affordable, and evidence-based mental health services in the United States and globally [1, 2]. More than 264 million people around the world were affected by depression in 2017 and the rate of depressive symptoms has continued to rise dramatically since the onset of the COVID-19 pandemic [3, 4]. The alarming gap between the number of individuals who need care and the supply of providers in the current behavioral health workforce has resulted in a large unmet need for mental health treatment [5]. The lack of face-to-face treatment options available during the pandemic further exacerbated existing barriers to care, such as cost, stigma, and an overreliance on one-on-one therapy with licensed providers [6]. Collectively, this has created an overwhelming need to expand and better allocate evidence-based mental health services to fill serious gaps in population-level health care access.

Digitally delivered mental health services that leverage videoconferencing technology have emerged as a scalable option to meet this demand [7]. Technology-enabled mental health care that delivers virtual one-on-one support appears to produce similar outcomes as in-person treatment [8, 9] and offers several promising solutions to enhance care. Digital platforms that collect information before treatment can use patient characteristics and preferences to identify and deliver the most effective yet least burdensome and least costly intervention from a range of care modalities, including psychotherapy with paraprofessionals or licensed therapists [10]. Research has shown that this initial allocation of services is both clinically and cost-effective [11–14] and given global behavioral health workforce gaps [5], connecting people for whom it is indicated with providers like certified professional coaches will help expand access to high-quality, affordable care [15, 16]. Considering these potential benefits, more research is warranted to investigate digital mental health services that leverage technology to offer personalized recommendations and deliver care with different types of providers.

In the present study, we examined clinical and cost outcomes from a digital platform that recommends care and delivers one-on-one virtual mental health services. The platform uses brief psychological assessments to recommend adults with symptoms of depression to telecoaching or teletherapy services. We analyzed data from participants who registered for care during the first year of the COVID-19 pandemic (March 2020—March 2021) and screened positive for depressive symptoms. We had three aims: 1) evaluate changes in subjective well-being and rates of clinical improvement from depressive symptoms overall and among the different utilization patterns, 2) investigate associations between utilization patterns and the likelihood of clinical improvement in depressive symptoms, and 3) compare estimated costs between utilization patterns.

## Method

### Design and participants

We analyzed retrospective de-identified data from participants who registered for mental health services through a digital platform (Modern Health, Inc., San Francisco, CA) during the COVID-19 pandemic (3/11/2020–3/11/2021). Eligible participants were: 18 years or older; had access to a smartphone, tablet, or computer; screened positive for depressive symptoms (World Health Organization-Five [WHO-5] Well-Being Index ≤ 28) at registration; and completed a follow-up assessment at least 14 days after registering. Western Clinical Group IRB reviewed this study and determined it to be exempt from IRB oversight.

### Procedures

Eligible adults registered on the web or mobile application using a device (smartphone, tablet, or computer). As part of their registration, participants completed a baseline questionnaire, where they selected topics and symptoms of concern from five areas (emotional, professional, physical, social, and financial health), indicated their preferences for care modality, and completed the WHO-5. Participants were recommended a care plan based on their initial clinical acuity and care preferences. Though a care plan was recommended, participants could self-refer and utilize combinations of teletherapy and telecoaching, as well as digital resources and assessments. There were no prescribed number of teletherapy or telecoaching visits to complete and participants could utilize visits, digital resources, and assessments at their discretion within the limits of the plan offered by their employer. Participants who utilized teletherapy or telecoaching could message with their provider between sessions and could rate their satisfaction with the provider after each visit. Participants were prompted to voluntarily complete a follow-up assessment at least 14 days after registering through Modern Health’s secure platform.

### Evidence-based digital mental health services

The platform offers several evidence-based modalities of care, including one-on-one care with mental health professionals. In this study, participants had access to teletherapy, telecoaching, or both. Below are descriptions of the teletherapy and telecoaching programs.

#### Teletherapy

The platform has a global network of high-quality providers who practice evidence-based care. Therapists were licensed providers with advanced degrees in clinical psychology or a related field (e.g., Doctor of Philosophy, Doctor of Psychology, Licensed Clinical Social Worker, Licensed Marriage and Family Therapist, Licensed Professional Counselor), with advanced training and practice in evidence-based care (e.g., CBT, Acceptance and Commitment Therapy [ACT], Dialectical Behavior Therapy, Interpersonal Therapy), and demonstrated alignment with short-term, evidence-based, culturally responsive, and ethical care. Therapy sessions lasted approximately 50 minutes each, and were most often scheduled to occur weekly, but could be spaced out according to the individual’s needs. Therapy visits were provided via videoconferencing through the digital platform. The number of therapy sessions an individual utilized could vary based on the number of covered sessions offered by their employer, their clinical needs, and their engagement with treatment.

#### Telecoaching

All coaches were certified by an International Coaching Federation (ICF) accredited program and screened and vetted to ensure they were trained in and offered evidence-based approaches (primarily non-clinical techniques that draw from the principles of CBT, ACT, Motivational Interviewing, and Mindfulness-Based Approaches). Coaching sessions lasted approximately 30 minutes each and were provided via videoconferencing. Coaches identified goals the participant wanted to work towards, used evidence-based principles like CBT or ACT to help the participant think about self-beliefs or behaviors impeding on those goals, and explored action plans for the participant to work towards desired outcomes. To ensure ongoing quality, clinical care managers collected and reviewed aggregate feedback, and offered ongoing training and case consultation to support all coaches and therapists.

### Measures

Standardized and validated symptom questionnaires are administered to participants at the baseline assessment and throughout treatment. The WHO-5 questionnaire is the primary treatment outcome measurement [17].

#### Utilization pattern

We characterized utilization patterns by the types of services an individual had engaged over the study period. In this study, we classified individuals as having utilized: 1) assessments only, 2) telecoaching only (at least 1 session), 3) teletherapy only (at least 1 session), or 4) both telecoaching and teletherapy (at least 1 session of each type of care).

#### Subjective well-being and depressive symptoms

We used the WHO-5 to assess well-being and depressive symptoms within the past two weeks on a six-point scale (0 = *at no time*, 5 = *all of the time*), which is a unidimensional assessment of well-being with high clinimetric validity as a screening tool for depression [17]. Scores are summed and multiplied by 4, with higher scores indicating greater well-being and lower depressive symptomatology. Clinical improvement in depressive symptoms is defined as an increase of at least 10 points and clinical deterioration is defined as a decrease of at least 10 points [17, 18].

#### Cost of care

We conservatively estimated an hourly rate for coaches and therapists based on the national 2021 Medicare reimbursement rate of $103.28 for a 50 minute psychotherapy session [19]. Extrapolating from this rate, we modeled the adjusted cost to $61.97 per telecoaching session since coaching sessions are approximately 40% shorter on average (30 vs. 50 minutes). The cost of care for each individual was estimated as the sum of the product of the cost of the actual telecoaching and/or teletherapy sessions utilized during the study period.

#### Satisfaction with care

Satisfaction ratings can serve as a proxy for treatment acceptability and therapeutic alliance with providers. For participants who utilized teletherapy and/or telecoaching, we assessed satisfaction with a 5-star rating. Modern Health prompts individuals to rate their satisfaction after every visit.

### Data analysis

We conducted analyses using R version 4.0.4. We used two-sided paired t-tests to evaluate differences in subjective well-being between baseline and follow-up assessments in our overall sample, as well as stratified by utilization patterns. Due to a strongly skewed distribution, we performed two-sided Kruskal-Wallis tests to evaluate differences in the cost of care between telecoaching, teletherapy, and having utilized both services. Post hoc Dunn tests were additionally performed to evaluate which utilization patterns differed significantly in terms of cost of care. We constructed logistic regression models to estimate the association between utilization patterns and clinical improvement given the binary nature of clinical improvement in depressive symptoms. We focused on clinical improvement in depressive symptoms (i.e., a 10-point increase at follow-up) as opposed to clinical recovery (i.e., moving above the clinical cut-off of 28) because it is a more robust outcome and a more challenging metric to achieve in the overall sample (e.g., some participants starting care with a WHO-5 score of 28 only need to increase one point to achieve recovery). The adjusted regression model included age, sex, baseline WHO-5 score, and number of days between baseline and follow-up assessments (to control for time) as covariates. We considered hypothesis tests statistically significant using an alpha level of 0.05.

## Results

### Study participants

Of the 7,476 adults who initially screened positive for depression at baseline during the study period, 1,852 (24.8%) had a follow-up assessment at least 14 days after baseline available for analysis. The average age of participants was 35.1 years (SD = 8.5, range = 20–71), 57.2% identified as female and 26.6% identified as male. We were missing sex from 16.1% of the sample due to optional reporting from employers. The mean WHO-5 score of participants at baseline was 21.1 (SD = 6.5, range = 0–28) and 31.2 (SD = 17.7, range = 0–100) at their last available follow-up assessment. The average time between baseline and follow-up assessments was 158 days (SD = 111, range = 14–467). Among those who had utilized care with a provider, the average time spent in care was 69 days (SD = 87, range = 0–449) and the average time between their last session and follow-up assessment was 75 days (SD = 95, range = 0–452). See Table 1 for the demographic and clinical factors in the overall sample and utilization-stratified samples.

**Table 1:** Descriptives of demographic and clinical factors among registrants of a digital mental health platform.

	Utilization				
	Assessments Only (n = 758)	Telecoaching (n = 401)	Teletherapy (n = 455)	Telecoaching and Teletherapy (n = 238)	Total (n = 1852)
**Demographic Factors**					
**Age**					
Mean	35.78	35.20	34.38	34.24	35.11
SD	9.13	8.38	8.06	7.03	8.48
**Sex**					
Female	403 (53.2%)	251 (62.6%)	252 (55.4%)	154 (64.7%)	1060 (57.2%)
Male	230 (30.3%)	94 (23.4%)	123 (27.0%)	46 (19.3%)	493 (26.6%)
Unknown	125 (16.5%)	56 (14.0%)	80 (17.6%)	38 (16.0%)	299 (16.1%)
**Clinical Factors**					
**Baseline Subjective Well-being (WHO-5)**					
Mean	21.59	22.26	19.46	20.69	21.09
SD	6.16	6.34	6.98	6.40	6.52
**Follow-up Subjective Well-being (WHO-5)**					
Mean	27.43	35.60	31.93	34.40	31.20
SD	16.12	17.76	18.33	18.58	17.68
**Follow-up Period (Number of days between baseline and follow-up assessment)**					
Mean	128.86	146.10	179.99	227.23	157.80
SD	97.61	104.12	113.44	115.53	110.54
**Difference in Baseline and Follow-up WHO-5**					
Mean	5.84	13.34	12.48	13.71	10.10
SD	15.92	17.85	18.42	19.00	17.74
**Clinical Improvement**	241 (31.8%)	210 (52.4%)	225 (49.5%)	123 (51.7%)	799 (43.1%)

*Note*. WHO-5 = World Health Organization-Five Well-Being Index.

### Utilization

The most common utilization pattern among those with a positive depression screening at baseline was assessments only (40.9%), followed by teletherapy (24.6%), telecoaching (21.7%) and both services (12.9%). Individuals who utilized telecoaching alone completed an average of 3.2 sessions (SD = 3.3, range = 1–26), while those who utilized teletherapy alone completed an average of 5 sessions (SD = 5.1, range = 1–40). Individuals who utilized both telecoaching and teletherapy completed an average of 3.5 telecoaching sessions (SD = 3.2, range = 1–24) and 5.1 teletherapy sessions (SD = 4.1, range = 1–22). The majority of those utilizing both services started with telecoaching (73.1%), while 25.6% started with teletherapy and 1.3% started both services on the same day.

### Subjective well-being

Regarding changes in subjective well-being between baseline and follow-up in our overall sample (see Table 2), paired t-tests found that participants reported an average improvement of 10.1 points on the WHO-5, which represents a 48% increase and medium effect size (*d* = 0.73). Those who utilized assessments only reported an average increase of 5.8 points, representing a 27% improvement and small effect size (*d* = 0.46). Those who utilized telecoaching alone reported an average increase of 13.3 points, which represents a 60% improvement and a large effect size (*d* = 0.97). Those who utilized teletherapy alone reported an average increase of 12.5 points, which represents a 64% improvement and a large effect size (*d* = 0.87). Those who utilized both telecoaching and teletherapy reported an average increase of 13.7 points, representing a 66% improvement and a large effect size (*d* = 0.97).

**Table 2:** Two-tailed paired t test results for subjective well-being outcomes.

		Baseline	Follow-up	Paired Pre-Post Difference	CIs of Pre-Post Difference		
*Sample*	*n (%)*	*M* (*SD*)	*M* (*SD*)	*M*	95% CIs	*t (df)*	*p*
**Overall Sample**							
Full	1852 (100%)	21.09 (6.52)	31.20 (17.68)	10.10	9.3–10.9	24.51 (1851)	< .001
**Utilization Type**							
Assessments Only	758 (40.9%)	21.59 (6.16)	27.43 (16.12)	5.84	4.7–6.97	10.09 (757)	< .001
Telecoaching	401 (21.7%)	22.26 (6.34)	35.60 (17.76)	13.34	11.6–15.1	14.96 (400)	< .001
Teletherapy	455 (24.6%)	19.46 (6.98)	31.93 (18.33)	12.47	10.8–14.2	14.45 (454)	< .001
Telecoaching & Teletherapy	238 (12.9%)	20.69 (6.40)	34.40 (18.58)	13.71	11.3–16.1	11.13 (237)	< .001

*Note*. *SD* = standard deviation, CI = 95% confidence intervals.

### Clinical improvement

As shown in Table 3, unadjusted logistic regression results found that participants who utilized any provider care were significantly more likely to achieve clinical improvement in depressive symptoms at follow-up. After adjusting the model for age, sex, baseline WHO-5 score, and number of days between baseline and follow-up assessments, those who utilized telecoaching (aOR = 2.45, 95% CI: 1.91–3.15, p < 0.001), teletherapy (aOR = 2.01, 95% CI: 1.57–2.57, p < 0.001), or both services (aOR = 2.28, 95% CI: 1.67–3.11, p < 0.001) were significantly more likely to achieve clinical improvement than those who only used assessments. In the overall sample, 9.3% (n = 173) of participants clinically deteriorated. The majority (51%) of those who deteriorated were participants who utilized assessments only (n = 88). Participants who clinically deteriorated (M = 24.53, SD = 4.11) had higher average baseline WHO-5 scores than those who did not clinically deteriorate (M = 20.74, SD = 6.62), t(1850) = -7.39, p < .001, Cohen d = 0.59.

**Table 3:** Unadjusted and adjusted logistic regression results for clinical improvement by utilization type.

	Clinical Improvement						
		Unadjusted model			Adjusted Model (a)		
	n (%)	OR	95% CI	p	aOR	95% CI	p
(Intercept)		0.47	0.40–0.54	< .001	0.60	0.34–1.05	.07
Age	--	--	--	--	1.00	0.99–1.02	.43
Sex	--	--	--	--	--	--	--
Female (reference)	--	--	--	--	--	--	--
Male	--	--	--	--	1.16	0.93–1.45	.20
Unknown	--	--	--	--	1.21	0.93–1.58	.15
Baseline WHO-5 Score	--	--	--	--	0.98	0.96–0.99	.001
Follow-Up Time	--	--	--	--	1.00	0.99–1.00	.71
Utilization Pattern	--	--	--	--	--	--	--
Assessments Only (reference)	241 (31.8%)	--	--	--	--	--	--
Telecoaching	210 (52.4%)	2.36	1.84–3.03	<0.001	2.45	1.91–3.15	<0.001
Teletherapy	225 (49.5%)	2.1	1.65–2.67	<0.001	2.01	1.57–2.57	<0.001
Telecoaching & Teletherapy	123 (51.7%)	2.29	1.71–3.09	<0.001	2.28	1.67–3.11	<0.001

*Note*. *n* = 1,852. WHO-5 = World Health Organization-Five Well-Being Index. OR = Odds ratio; CI = Confidence interval; Follow-Up Time = Number of days between baseline and follow-up assessments.

(a) Adjusted model additionally included covariates.

Unadjusted model: Goodness of Fit (Hosmer and Lemeshow): X-squared: 2.4094e-23, df = 8, p-value = 1. Adjusted Model: Goodness of Fit (Hosmer and Lemeshow): X-squared: 6.3826, df = 8, p-value = .60.

### Cost of care

The median total cost of care based on Medicare reimbursement rates was $124, $413, and $559 among those utilizing telecoaching, teletherapy, and both teletherapy and telecoaching, respectively. There was a significant difference in the median cost of care by utilization pattern (Kruskal-Wallis chi-square = 393.17, p < .001), and a post hoc Dunn test found that the cost of all utilization patterns differed significantly from each other. Specifically, telecoaching was less costly than teletherapy (Z = -14.14, p < .001) and both services (Z = 18.26, p < .001), and teletherapy was less costly than both services (Z = 6.57, p < .001).

### Satisfaction with care

Among those who had utilized care with a provider, 53.6% (n = 586) completed at least 1 satisfaction rating following a session. Average satisfaction was 4.86 (SD = 0.48) and similar among those who utilized telecoaching (M = 4.87, SD = 0.48), teletherapy (M = 4.84, SD = 0.58), and both telecoaching and teletherapy (M = 4.86, SD = 0.35).

## Discussion

We examined relative changes in subjective well-being and depressive symptoms and compared the costs between utilization patterns from a technology-enabled platform that delivers digital mental health services. We found that individuals who had originally reported elevated depressive symptoms at intake reported significant improvements in subjective well-being and clinically meaningful changes in depressive symptoms at follow-up. Utilization of telecoaching, teletherapy, or both services were similarly associated with greater clinical outcomes than having only utilized assessments during the study period and telecoaching was found to be the most economical utilization pattern.

In our sample of individuals who had registered for the platform during the COVID-19 pandemic, subjective well-being increased by an average of 10 points at follow-up, which represents both a statistically and clinically significant improvement. Larger effective sizes were observed among those who had utilized telecoaching, teletherapy, or both services compared to those utilizing assessments only. In a recent systematic review and meta-analysis evaluating the effectiveness of interventions on subjective well-being among working populations, psychosocial interventions were found to be the most effective in improving subjective well-being among working populations [20]. Although subjective well-being scores significantly increased post-intervention, the average well-being score was still relatively low (31/100) yet was above the threshold for moderate to severe depressive symptoms. A commonly used cut-off indicating mild or no depressive symptoms on the WHO-5 is a score greater than or equal to 50 [17]. Achieving this level of average improvement may occur with more time as more participants utilize the mental health services and future interventions should focus on increasing utilization and uptake. Nevertheless, our findings suggest that psychosocial interventions for subjective well-being remain effective under real world conditions outside of randomized controlled trial conditions.

Additionally, 43.1% of those who initially reported depressive symptoms showed clinical improvement at follow-up. Previous research has similarly found that digitally delivered mental health services can be effective in fostering clinical improvement from depression. A recent systematic review that included both controlled and uncontrolled studies of mental health systems that recommended care via pre-defined decision criteria found that recovery rates for depression in working age adults was between 40–60% [11]. Our results suggest that digital mental health services delivering care in this way may be similarly effective, especially given the more robust threshold of clinical improvement we tracked in our study (i.e., increasing 10 points) compared to clinical recovery (i.e., moving above a cut-off).

We also found that those utilizing telecoaching, teletherapy, or both services were significantly more likely to report clinical improvement in depressive symptoms at follow-up than individuals who had only utilized assessments. The observed effect sizes were similar across telecoaching, teletherapy, and having utilized both types of services. Previous research has similarly found low intensity psychosocial interventions may produce similar clinical benefits, [21] even for those with higher initial severity in depressive symptoms [22]. England’s Improving Access to Psychological Therapies programme that similarly recommends and delivers mental health treatment using patient characteristics recently reported that progressive delivery of services beginning with low intensity psychological well-being practitioners was associated with better clinical outcomes than stratified care that began with higher intensity therapists [23]. Finally, we found that 11.6% of participants who utilized assessments only clinically deteriorated and 7.8% of participants who utilized any one-on-one care deteriorated. Although few studies (around 6%) report deterioration rates [24], some prior work has found that rates vary between 0–25% in psychotherapy treatment groups and 11–44% in comparison groups [24–27]. The deterioration rates we observed are on the low end of these ranges and appear reasonable given this study was not a controlled trial and took place during the COVID-19 pandemic when mental health concerns have risen.

While the observed clinical outcomes and satisfaction were similar across utilization patterns that involved a provider, the average cost of care per individual varied significantly, such that telecoaching was the most economical service followed by teletherapy and the combination of telecoaching and teletherapy. The incorporation of telecoaching into digitally delivered mental health care models may improve their cost-effectiveness compared to dominant treatment models that only provide in-person psychotherapy. A previous randomized trial in a primary care setting similarly suggests that recommending services as a function of patient clinical symptoms may be more cost-effective for the treatment of depression than treatment as usual [13]. Future research is needed to evaluate the cost-effectiveness of digital mental health services to optimize use of limited resources and ultimately improve access to care.

In our sample, we found that 40.9% of registrants had not initiated digital care with either a coach or therapist by the follow-up assessment. Previous research has found similar rates of engagement with care in both digital and in-person mental health services as the majority of those with depression remain untreated. The national MindSpot Clinic in Australia, which provides digital mental health services, found the majority (67%) of patients reported their main purpose of using Mindspot was for assessments and information, while only 25.9% reported seeking treatment [14]. A large-scale retrospective analysis of electronic medical records in the United States found that only 35.7% of patients newly diagnosed with depression in primary care initiated treatment [28], which suggests that the delivery of multiple digital mental health services in this study may have helped to improve treatment initiation rates compared to usual care settings. Furthermore, emerging evidence suggests that digital modalities of care, such as iCBT that may or may not involve a provider, can be effective treatments and may be an important modality for reducing the proportion of individuals with mental health conditions that do not initiate treatment [29].

There are several important limitations to consider when interpreting the results of our study. Most notably, our analysis was limited to individuals who had completed a follow-up assessment during the study period. Although missing data is common in routine cohorts outside of trial environments, the exclusion of individuals without follow-up data introduces the potential that our results may not generalize to all individuals who screened positive for depressive symptoms at baseline [30]. Our study was also observational in nature and our findings require experimental confirmation to directly attribute clinical outcomes with the intervention or directly estimate the relative effectiveness of telecoaching, teletherapy, and having utilized both services. Despite these limitations, our study also has notable strengths. The longitudinal data analyzed in this study was collected under real world conditions, which may improve the generalizability of results compared to data collected in controlled trial conditions. Additionally, the average follow-up assessment occurred more than 2 months after the final visit for those who had utilized care with a provider, suggesting that benefits may be sustained.

## Conclusions

This study demonstrates that digital mental health services including telecoaching and teletherapy were effective in improving subjective well-being and clinical improvement in depressive symptoms. While the likelihood of clinical improvement was similar across services, the average cost of care was significantly less for telecoaching. Digitally delivered mental health services may be a promising means of achieving clinical and cost-effective outcomes for those experiencing depressive symptoms.
